# Healthful and unhealthful provegetarian food patterns and micronutrient intake adequacy in the SUN cohort

**DOI:** 10.1017/S136898002200204X

**Published:** 2023-03

**Authors:** Daniela Asfura-Carrasco, Susana Santiago, Itziar Zazpe, Clara Gómez-Donoso, Maira Bes-Rastrollo, Miguel Ángel Martínez-González

**Affiliations:** 1 Department of Nutrition and Food Sciences and Physiology, University of Navarra, Pamplona, Spain; 2 Department of Preventive Medicine and Public Health, School of Medicine–Clínica Universidad de Navarra, University of Navarra, Irunlarrea 1, 31008 Pamplona, Navarra, Spain; 3 CIBER Fisiopatología de la Obesidad y Nutrición (CIBERObn), Instituto de Salud Carlos III, Madrid, Spain; 4 Navarra Institute for Health Research (IdiSNA), Pamplona, Spain; 5 Department of Nutrition, Harvard T.H. Chan School of Public Health, Boston, MA, USA

**Keywords:** Mediterranean cohort, plant-based diet, micronutrients intake adequacy, provegetarian food pattern

## Abstract

**Objective:**

To investigate the association between different versions of a provegetarian food pattern (FP) and micronutrient inadequacy.

**Design:**

Cross-sectional analysis. Dietary intake was assessed at baseline through a validated 136-item FFQ. Participants were classified according to groups of different versions of a provegetarian FP: overall, healthful and unhealthful. The prevalence of inadequate intake of vitamins B_1_, B_2_, B_3_, B_6_, B_12_, C, A, D, E, folic acid, Zn, I, Se, Fe, Ca, K, P, Mg and Cr was evaluated using the estimated average requirement (EAR) cut-point method and the probabilistic approach. Logistic regression analyses were conducted to estimate the probability of failing to meet EAR for either ≥ 3 or ≥ 6 micronutrients.

**Setting:**

Seguimiento Universidad de Navarra (SUN) cohort.

**Participants:**

17 825 Spanish adults.

**Results:**

Overall, subjects in the highest group of the unhealthful provegetarian FP had the highest prevalence of inadequate dietary intake for every vitamin and mineral, compared to those in the lowest group. The adjusted OR of failing to meet ≥ 3 EAR (highest *v*. lowest group) was 0·65 (0·54, 0·69) for the overall, 0·27 (0·24, 0·31) for the healthful and 9·04 (7·57, 10·4) for the unhealthful provegetarian FP.

**Conclusion:**

A higher adherence to an overall and healthful provegetarian FP was inversely associated with the risk of failing to meet EAR values, whereas the unhealthful version was directly associated with micronutrient inadequacy. Provegetarian FP should be well planned, prioritising nutrient-dense plant foods and minimising ultra-processed and unhealthy ones.

The concept of plant-based diets (PBD) is used differently by researchers, and it has no specific definition^(1)^. In general, PBD provide the majority of energy from plant-based foods, including vegetables, wholegrains, legumes, nuts, seeds and fruits, with few or no animal products^(2)^. People choose PBD for a variety of reasons including concern about animal welfare, as well as health or environmental concerns^(3)^.

There are different types of PBD according to the degree of exclusion of animal products, ranging from strict veganism to several types of vegetarianism^(4)^. In recent years, PBD have become more popular worldwide. While vegan or highly restrictive vegetarian diets are not likely to be easily adopted by the general population^(5–8)^, provegetarian or flexitarian food patterns (FP) may be a better alternative to reduce the consumption of animal foods and achieve long-term sustainable adherence to PBD.

The provegetarian or flexitarian diet is a flexible dietary pattern style that prioritises the consumption of plant or plant-based foods and beverages and incorporates animal foods (dairy products, eggs, meats and fish) less frequently and/or in smaller portions. The overall provegetarian FP was calculated according to the score proposed by Martínez-González *et al.* ^(9)^, which quantifies the habit of preferentially consuming plant-derived foods instead of animal-derived foods without the need to follow a strict vegetarian diet.

The PBD, if well planned, can support healthy nutrition at every age and life stage in healthy subjects and contribute to preserving the environment^(10)^. In fact, the 2020 Dietary Guidelines Advisory Committee Scientific Report recommends the promotion of PBD for a better health, among other dietary patterns^(11)^. However, previous studies have indicated that PDB do not necessarily imply a high nutritional quality and could be associated with higher cardiometabolic risk, especially if the plant foods included are highly processed and not very healthy or if the excluded food groups are of high nutritional density^(12)^. It is also worth mentioning that very restrictive PBD might present an increased risk of nutritional deficiencies and could seriously affect health^(6,13)^. Additionally, supplementation of specific nutrients such as I, Ca and vitamins B_12_ and D should be considered in several risk groups that follow a PBD^(10,14,15)^.

In nutritional epidemiology, there is a great interest to know how certain PBD could potentially reduce diet-related chronic disease morbidity and mortality^(13)^. Previous cohort studies have consistently used different versions of a provegetarian FP, including healthful and unhealthful ones^(9,14)^, to explore their relationship with chronic conditions. These healthy and unhealthy versions follow the scoring criteria suggested by Satija et al. for type 2 diabetes^(16)^. These patterns were based on the classification of plant-derived foods in two groups: healthy (fruits, vegetables, wholegrains, nuts, legumes, olive oil and coffee) and less-healthy (fruit juices, potatoes, refined grains, pastries and sugary beverages). Evidence to date has shown that the overall and healthful provegetarian FP reduce the risk of all-cause mortality^(9,14)^ and might decrease the risk of breast cancer^(15)^, overweight/obesity^(17)^ and cardiometabolic diseases^(13,16)^. Nevertheless, there is no evidence on the association between the provegetarian FP indices and micronutrient adequacy. Our hypothesis was that higher adherence to both overall and healthful provegetarian FP would be associated with higher micronutrient adequacy, whereas a higher adherence to an unhealthful provegetarian FP would be associated with lower nutritional adequacy. Thus, the aim of this study was to investigate the association between different versions of a provegetarian FP and nutritional adequacy considering nineteen micronutrients in the ‘Seguimiento Universidad de Navarra’ [University of Navarra Follow-up] (SUN) cohort study.

## Methods

### Design

The SUN Project (http://medpreventiva.es/MvbqgK) is a dynamic prospective cohort study of university graduates conducted in Spain since December 1999. Baseline assessment and follow-up information is gathered biennially by mail or web-based questionnaires. Self-administered questionnaires include information on sociodemographic, medical, lifestyle and dietary variables. The overall retention in the cohort exceeds 90 %. Additional details on its objectives, design and methods can be found elsewhere^(18)^.

### Subjects

Up to December 2019, 22 894 subjects had completed the baseline questionnaire of the SUN Project. Participants who were outside the predefined limits for energy intake, as proposed by Willett (< 3347·2 kJ/d or > 16 736 kJ/d for men and < 2092 kJ/d or > 14 644 kJ/d for women)^(19)^, were excluded (*n* 2169). Subjects whose intakes were outside the predefined intake values of any micronutrient (≥ 3 sd from both sides of the mean) were also excluded (*n* 2900). Finally, 17 825 participants were included in the analyses for the present study.

### Dietary assessment

Dietary intake was assessed at baseline using a 136-item semi-quantitative FFQ repeatedly validated in Spain^(20–22)^. The FFQ collected typical food intake over the previous year. A typical portion size was specified for each item, and consumption frequencies were registered in nine categories that ranged from ‘never or almost never’ to ‘≥ 6 times/day’. Daily intake (g/d) was calculated by multiplying the specified portion size of each food item by the frequency of consumption. A trained dietitian updated the nutrient database using the latest available information in the Spanish food composition tables.

### Exposure assessment – provegetarian food patterns

Provegetarian FP are based on gradual dietary changes, progressively increasing the consumption of plant-based foods and simultaneously reducing animal foods^(9,16)^. Specifically, the overall score quantifies the consumption (g/d) of seven plant food groups (fruits, vegetables, potatoes, nuts, legumes, cereal grains and olive oil) and five animal food groups (dairy products, eggs, meat, fish and seafood and animal fat). Quintile values of plant foods and reverse quintiles values of animal foods were summed; therefore, final scores can range from 12 (lowest adherence) to 60 points (highest adherence) (Table 1).

**Table 1:** Scoring criteria for the provegetarian food patterns

Overall provegetarian FP (potential range 12–60)		Healthful/unhealthful provegetarian FP (potential range of 18–90)		
Component	Criteria	Component	Criteria	
			Healthful	Unhealthful
**Plant food groups**		**Plant food groups**		
		***Healthful***		
1. Vegetables	Positive	1. Vegetables	Positive	Reverse
2. Fruits	Positive	2. Fruits	Positive	Reverse
3. Legumes	Positive	3. Legumes	Positive	Reverse
4. Cereal grains	Positive	4. Wholegrains	Positive	Reverse
5. Potatoes	Positive	5. Nuts	Positive	Reverse
6. Nuts	Positive	6. Olive oil	Positive	Reverse
7. Olive oil	Positive	7. Coffee	Positive	Reverse
		***Less-healthful***		
**Animal Food Groups**		8. Fruit juices	Reverse	Positive
8. Dairy products	Reverse	9. Potatoes		Positive
9. Eggs	Reverse	10. Refined grains	Reverse	Positive
10. Meat	Reverse	11. Sugary beverages	Reverse	Positive
11. Fish and seafood	Reverse	12. Pastries	Reverse	Positive
12. Animal fat	Reverse			
		**Animal Food Groups**		
		13. Dairy	Reverse	Reverse
		14. Eggs	Reverse	Reverse
		15. Meat	Reverse	Reverse
		16. Fish and seafood	Reverse	Reverse
		17. Miscellaneous food	Reverse	Reverse
		18. Animal fat	Reverse	Reverse

Miscellaneous food includes pizza, instant soups and mayonnaise.

As shown in Table 1, healthy (fruits, vegetables, wholegrains, nuts, legumes, olive oil and coffee) and less-healthy plant foods (fruit juices, potatoes, refined grains, pastries and sugary beverages) were distinguished for the other versions. For the healthful provegetarian FP, positive scores were assigned to healthy plant foods and reverse scores to less-healthy plant foods as well as to animal foods. In contrast, for the unhealthful provegetarian FP, positive scores were assigned to less-healthy plant foods and reverse scores to healthy plant foods and animal foods. Quintiles and reverse quintiles were summed to obtain scores of the healthful and unhealthful versions of a provegetarian FP. Thus, final scores could range from 18 (lowest adherence) to 90 (highest adherence).

### Outcome assessment – micronutrient adequacy

The total micronutrient intake was calculated by adding the average micronutrient intake from foods, beverages and dietary supplements. We assessed micronutrient intake adequacy taking into account the following nineteen micronutrients with known public health relevance: vitamins B_1_, B_2_, B_3_, B_6_, B_12_, C, A, D, E, folic acid, Zn, I, Se, Fe, Ca, K, P, Mg and Cr. When the specific estimated average requirement (EAR) value for a nutrient could not be determined, the adequate intake was used as the reference. Inadequate intake was defined as any micronutrient intake below the EAR if available or the adequate intake if EAR values were not available. Both dietary reference intakes have been proposed by the Institute of Medicine^(23)^. Nutrient intake adequacy for sixteen micronutrients (all except K and Cr because they have no EAR values, and Fe because of its skewed distribution) was also evaluated using the probabilistic approach, which calculated the probability of adequacy for a nutrient’s usual intake as follows: Z score = (estimated nutrient intake – EAR)/SD of the EAR. The Z scores correspond to an estimated probability of inadequacy according to normal distribution. Because of the skewed distribution of Fe intake, its value was log-transformed for the present study.

### Assessment of other variables

Information on non-dietary variables was also collected at baseline (e.g. medical history, sociodemographic characteristics, lifestyle and health-related habits). Self-reported data, such as physical activity^(24)^, BMI^(25)^ or hypertension^(26)^, have been previously validated in a subsample of the cohort. Three previously defined scores were also used to describe the baseline characteristics of participants: Carbohydrate Quality Index (range, 4–20)^(27,28)^, Fat Quality Index (range, 0·62–5·92)^(27,28)^ and the Mediterranean diet score (range, 0–9) developed by Trichopoulou *et al.* ^(29)^.

### Statistical analyses

Participants were categorised into the following three groups to create three reasonably equal groups in each provegetarian FP: [lowest adherence (overall provegetarian FP from 12 to 35, healthful from 30 to 52 and unhealthful from 31 to 55), medium adherence (overall provegetarian FP from 36 to 39, healthful from 53 to 58 and unhealthful from 56 to 60) and highest adherence (overall provegetarian FP from 40 to 57, healthful from 59 to 82 and unhealthful from 61 to 83)] according to their adherence to each of the provegetarian FP indices described above.

Baseline characteristics of participants as well as their baseline food consumption and energy and nutrient intakes were reported according to extreme groups of adherence to each provegetarian FP. The descriptive results are presented as mean and standard deviation or percentages (%) for quantitative variables and categorical variables, respectively^(30)^. The baseline prevalence of inadequate intake of each micronutrient (i.e. intake below EAR) according to groups of each provegetarian FP was also estimated.

Non-conditional logistic regression models were used to evaluate the relationship of each provegetarian FP and the risk of micronutrient inadequacy using the EAR cut-point method and the probabilistic approach. In all analyses, the lowest group was used as the reference category. Crude and multivariable-adjusted OR and its 95 % CI were estimated for two different outcomes: failing to meet EAR for either ≥ 3 or ≥ 6 micronutrients.

One multivariable-adjusted model was fitted for each provegetarian FP controlling for the following potential confounding factors: age (continuous), sex, supplement consumption (yes/no) and total energy intake (continuous). We did not control for education level, smoking, physical activity or previous weight change, because there is no convincing association with micronutrient adequacy (see online supplementary material, Supplemental Figure 1). Linear trend tests were performed through groups of each provegetarian FP by assigning the median score values of each group to participants and treating the variables as continuous. In addition, ANCOVA tests were performed to estimate the average number of micronutrients with intakes below the EAR across groups adjusting for sex and age.

Finally, sensitivity analyses were carried out to assess the robustness of the findings, excluding participants outside the 1st and 99th percentile of energy intake in one case and outside the 5th and 95th percentile in another. Additionally, analyses were performed excluding those with no answer in ≥ 30 items in the 136-item baseline FFQ.

Statistical analyses were carried out using STATA version 14 (STATA Corporation). All *P* values are two-tailed, and statistical significance was established in the conventional cut-off of *P* < 0·05.

## Results

The baseline characteristics of the 17 825 participants included in the study are summarised according to groups of the overall provegetarian FP in Table 2. Subjects with greater adherence to the overall provegetarian FP (third group, G3) were more likely to be more physically active and have a history of hypertension, CVD, diabetes, dyslipidaemia, cancer and hypercholesterolaemia. In addition, participants in the highest group of the overall provegetarian FP tended to have a higher consumption of dietary supplements, were more likely to follow special diets, and had a higher Mediterranean diet score^(29)^ and a higher Carbohydrate Quality Index and Fat Quality Index (reflecting higher dietary quality of carbohydrates and fat). On the other hand, participants with lower adherence to the overall provegetarian FP (first group, G1) were more likely to be active smokers (≥ 15 cigarettes/d), to snack between meals and to gain weight (≥ 3 kg) over the last 5 years.

**Table 2:** Baseline characteristics of participants according to adherence to the overall provegetarian FP: the Seguimiento Universidad de Navarra (SUN) cohort: 1999–2019

	Overall provegetarian FP									
	G1			G2			G3			*P* value
	%	Mean	sd	%	Mean	sd	%	Mean	sd	
Range	12–35			36–39			40–57			
*n*	7032			5452			5341			< 0·001
Age		36·2	11·7		37·8	12·0		40·0	12·9	< 0·001
Women (%)	57·3			61·4			61·5			< 0·001
BMI (kg/m^2^)		23·6	3·5		23·5	3·5		23·4	3·5	0·019
Physical activity (MET-h/week)		20·1	21·7		21·1	21·7		23·2	23·3	< 0·001
Sitting time (h/d)		5·4	2·1		5·3	2·1		5·2	2·0	< 0·001
Sedentary activities (h/d)		1·6	1·2		1·6	1·2		1·6	1·1	0·041
Smoke (%)										< 0·001
Never	48·4			47·9			47·5			
Former smokers	25·7			28·3			30·2			
Active smokers < 15 cigarettes/d	12·3			12·7			12·1			
Active smokers ≥ 15 cigarettes/d	11·0			8·8			7·4			
Prevalent hypertension (%)	10·2			10·0			12·4			< 0·001
Prevalent CVD (%)	1·3			1·3			2·1			< 0·001
Prevalent diabetes (%)	1·5			1·7			2·1			0·039
Prevalent dyslipidaemia (%)	6·1			6·8			7·7			0·002
Prevalent cancer (%)	1·9			2·7			2·8			0·002
Prevalent hypercholesterolaemia (%)	14·3			17·7			19·5			< 0·001
Family history of obesity (%)	19·2			20·0			21·6			0·005
Family history of CVD (%)	12·9			13·7			14·4			0·044
Marital status (%)										< 0·000
Single	48·6			44·6			41·9			
Married	46·7			49·8			51·9			
Other	4·7			5·6			6·2			
Supplements consumption (%)	14·6			17·2			17·5			< 0·001
Weight gain of ≥ 3 kg in the last 5 years (%)	32·8			30·2			26·9			< 0·001
Follow-up of special diet (%)	7·2			7·2			8·6			0·007
Snacking between meals (%)	35·6			33·0			31·2			< 0·001
Trichopoulou MedDiet score (range, 0–9)		3·2	1·5		4·3	1·5		5·5	1·5	< 0·001
Carbohydrate Quality Index (range, 4–20)		10·0	2·8		11·4	3·0		12·8	3·0	< 0·001
Fat Quality Index (range, 0·62–5·92)		1·5	0·3		1·7	0·4		2·0	0·5	< 0·001

MET, metabolic equivalents: FP, food pattern; G, groups.

Mean ± (sd) or %.

Food consumption according to extreme groups of adherence to each provegetarian FP is shown in Table 3. As expected, the consumption of fruits, vegetables, legumes, cereal grains, potatoes, fruit juices, olive oil and nuts increased across categories of the overall provegetarian FP, whereas the consumption of all animal food groups, coffee, sugar-sweetened beverages, sweets and desserts and miscellaneous food decreased. Moreover, participants with greater adherence to the healthful provegetarian FP had a higher consumption of fruits, vegetables, legumes, wholegrains, olive oil, nuts and coffee and a lower consumption of less healthy plant foods as well as all animal origin foods. By contrast, participants with greater adherence to the unhealthful provegetarian FP had a higher consumption of refined grains, potatoes, fruit juices, sugar-sweetened beverages and pastries, whereas they had a lower consumption of fruits, vegetables, legumes, wholegrains, olive oil, nuts and animal foods.

**Table 3:** Food consumption according to extreme groups of adherence to the overall, healthful and unhealthful provegetarian FP (Mean and sd)

	Overall provegetarian FP				Healthful provegetarian FP				Unhealthful provegetarian FP			
	G1		G3		G1		G3		G1		G3	
	Mean	sd	Mean	sd	Mean	sd	Mean	sd	Mean	sd	Mean	sd
Range	12–35		40–57		30–52		59–82		31–55		61–83	
*n*	7032		5341		6295		5860		6745		5770	
Fruits (g/d)	243·9	193·2	470·4	291·9	269·3	197·0	437·4	300·0	439·0	279·3	252·3	210·8
Vegetables (g/d)	393·7	230·1	635·5	283·7	404·2	225·5	604·5	294·9	633·8	280·2	354·4	210·1
Legumes (g/d)	18·6	15·5	27·3	18·9	20·9	15·3	24·5	20·3	26·5	20·3	18·0	13·8
Cereal grains (g/d)	76·4	57·5	123·9	68·5	107·5	67·4	90·6	63·8	96·6	61·3	101·5	72·4
Wholegrains (g/d)	7·0	18·7	18·5	35·0	4·1	14·4	22·8	36·9	20·5	34·2	4·1	16·1
Refined grains (g/d)	69·4	56·3	105·4	69·1	103·4	66·4	67·8	57·7	76·1	57·3	97·4	71·3
Potatoes (g/d)	42·8	38·8	66·2	47·7	69·1	47·6	37·6	35·5	48·3	41·6	59·2	46·3
Fruit juices (g/d)	55·3	95·4	71·8	99·1	70·2	95·0	53·5	89·8	59·3	85·9	66·2	106·5
Olive oil (g/d)	13·4	11·8	23·8	16·0	15·0	12·6	21·3	15·9	22·2	15·5	13·5	12·4
Nuts (g/d)	3·8	5·7	11·6	13·3	5·2	7·1	9·5	12·7	9·9	12·2	4·4	6·9
Dairy products (g/d)	429·2	210·3	327·1	187·5	432·7	203·7	332·0	200·0	430·3	210·2	331·7	191·3
Meat (g/d)	189·3	76·5	155·0	72·1	206·1	73·8	142·2	68·5	192·4	79·4	154·4	68·5
Eggs (g/d)	25·1	14·6	20·1	11·1	26·6	13·9	18·8	11·8	25·0	13·7	20·1	12·2
Fish and seafood (g/d)	95·3	51·8	93·7	52·6	94·5	50·9	94·2	53·8	115·6	52·9	71·6	42·4
Animal fat (g/d)	1·6	3·0	0·5	1·7	1·8	3·2	0·5	1·8	1·4	2·9	0·7	2·1
Coffee (g/d)	62·0	63·4	59·6	60·2	51·6	57·4	70·2	64·6	73·6	64·3	46·1	55·2
Sugar sweetened beverages (g/d)	69·6	111·2	54·1	96·3	97·8	126·5	30·5	72·3	45·2	85·9	84·9	123·0
Pastries (g/d)	50·2	44·7	47·8	42·5	64·2	44·9	34·6	37·5	42·6	37·6	56·8	49·2
Miscellaneous food (g/d)	23·9	36·6	20·6	35·9	34·4	42·6	11·6	25·1	25·5	39·6	18·9	31·7
Alcohol (g/d)	7·3	10·7	7·9	10·4	7·5	10·4	7·6	10·8	7·9	10·6	7·3	10·9

FP, food pattern; G, groups.

Miscellaneous food includes: pizza, instant soups and mayonnaise.

Energy and nutrients intake according to extreme groups of each provegetarian FP are shown in Table 4. The intake of total energy, carbohydrates, fibre, PUFA and *n*-3 fatty acids and all micronutrients except vitamin B_12,_ vitamin D, Ca and I were significantly greater in the group with the highest adherence to the overall provegetarian FP (G3 compared to G1). Conversely, the intake of protein, total fat, MUFA, SFA, TFA and cholesterol were significantly lower in G3 compared to G1.

**Table 4:** Energy and nutrient intakes according to extreme groups of adherence to the overall, healthful and unhealthful provegetarian FP (Mean and sd)

	Overall provegetarian FP				Healthful provegetarian FP				Unhealthful provegetarian FP			
	G1		G3		G1		G3		G1		G3	
	Mean	sd	Mean	sd	Mean	sd	Mean	sd	Mean	sd	Mean	sd
Range	12–35		40–57		30–52		59–82		31–55		61–83	
*n*	7032		5341		6295		5860		6745		5770	
Energy (kJ/d)	8983	2473·2	10 276	2353·1*	10 657	2350·2	8586	2312·5*	10 255	2341·8	8878	2492·0
Carbohydrates intake (% of total energy [E])	40·3	7·0	46·1	6·7*	42·8	6·4	43·5	7·9*	41·4	6·9	45·0	7·4
Fibre (g/d)	20·7	7·9	34·2	10·3*	23·3	8·6	30·8	11·6*	32·2	10·5	20·9	8·4*
Protein intake (% total E)	19·2	3·3	16·8	2·6*	17·7	2·7	18·4	3·4*	18·9	3·0	17·1	3·1*
Fat intake (% total E)	38·2	6·1	35·0	6·2*	37·5	5·5	35·7	7·0*	37·5	6·2	35·6	6·3*
PUFA intake (% total E)	5·1	1·4	5·2	1·4*	5·4	1·4	4·9	1·4*	5·1	1·2	5·3	1·6*
*n*-3 Fatty acids (g/d)	2·5	1·2	2·6		2·8	1·2	2·3	1·0*	2·9	1·0	2·2	1·2*
*n*-6 Fatty acids (g/d)	17·4	12·1	17·5	10·9	21·8	13·0	13·4	9·3*	17·5	10·2	17·4	13·0*
MUFA intake (% total E)	15·9	3·4	15·7	3·7*	15·5	3·0	16·0	4·2*	16·4	3·6	14·9	3·4*
SFA intake (% total E)	13·9	3·1	10·8	2·6*	13·6	2·8	11·2	3·0*	12·4	3·0	12·5	3·2*
TFA intake (% total E)	0·4	0·2	0·3	0·1*	0·4	0·2	0·3	0·2*	0·3	0·2	0·4	0·2*
Cholesterol (mg/d)	437·9	145·2	367·4	127·5*	478·4	134·4	332·5	115·7*	443·9	143·8	362·3	121·9*
Vitamin A (μg/d)	1482·8	919·5	2366·3	1225·9*	1561·2	929·7	2213·6	1257·9*	2352·9	1197·0	1342·0	875·6*
Vitamin E (mg/d)	5·8	2·6	7·7	3·1*	6·7	2·8	6·6	3·1*	7·6	2·9	5·5	2·6*
Vitamin C (mg/d)	213·7	107·0	335·3	133·3*	233·9	111·0	308·0	139·9*	325·3	131·9	208·3	108·6*
Vitamin B_1_ (mg/d)	1·7	0·6	2·0	0·7*	1·9	0·6	1·8	0·6*	2·1	0·6	1·6	0·6*
Vitamin B_2_ (mg/d)	2·1	0·6	2·2	0·6*	2·3	0·6	2·0	0·7*	2·4	0·6	1·9	0·6*
Vitamin B_3_ (mg/d)	40·7	11·3	42·6	10·9*	44·6	10·8	38·9	11·1*	47·2	10·4	35·6	9·8*
Vitamin B_6_ (mg/d)	2·5	0·8	3·1	0·9*	2·7	0·8	2·8	0·9*	3·2	0·8	2·3	0·8*
Vitamin B_12_ (mg/d)	9·7	4·3	8·6	4·1*	10·0	4·2	8·3	4·0*	10·7	4·3	7·5	3·6*
Vitamin D (μg/d)	6·1	3·9	6·1	3·9*	6·2	3·8	5·9	4·1*	7·4	4·2	4·8	3·2*
Folic acid (μg/d)	332·7	129·6	475·4	150·7*	358·8	132·4	440·4	161·5*	474·2	148·1	312·5	124·1*
Fe (mg/d)	15·1	4·9	19·2	5·2*	17·2	5·1	17·0	5·8*	19·2	5·2	14·6	4·8*
Ca (mg/d)	1145·5	390·2	1162·5	372·1*	1207·2	374·2	1099·7	387·7*	1312·9	376·0	977·4	337·6*
K (mg/d)	4002·9	1173·6	5277·8	1336·3*	4500·2	1228·4	4709·1	1453·4*	5302·0	1283·0	3801·5	1119·7*
Mg (mg/d)	354·9	95·0	459·2	107·2*	401·6	99·1	408·5	118·3*	460·4	102·3	339·8	92·1*
P (mg/d)	1818·1	465·4	1872·6	447·6*	1937·5	441·9	1758·4	465·6*	2109·3	419·3	1552·1	377·2*
Cr (μg/d)	74·8	30·7	94·6	31·8*	89·4	32·6	79·6	31·4*	90·9	31·0	76·1	33·1*
Se (μg/d)	89·2	29·9	95·4	29·7*	99·2	29·6	85·4	29·3*	103·0	28·3	80·3	29·0*
I (μg/d)	330·5	165·5	273·5	148·0*	330·9	159·3	275·7	156·0*	349·6	163·3	256·4	147·7*
Zn (mg/d)	15·6	7·1	17·1	7·1*	16·0	5·9	16·7	8·2*	18·8	7·5	13·6	6·0*

FP, food pattern; G, groups; TFA, trans fatty acids.

* *P* < 0·001 obtained through ANOVA test.

Participants with higher adherence to the healthful provegetarian FP had a higher intake of carbohydrates, fibre, protein and MUFA, and lower intake of energy, total fat, PUFA, *n*-3 and *n*-6 fatty acids, SFA, TFA, cholesterol, vitamins E, B_1_, B_2_, B_3_, B_12_ and D, Fe, Ca, P, Cr, Se and I compared to those with lower adherence (all these differences were statistically significant).

Finally, the intake of energy, fibre, protein, fat, MUFA and *n*-3 fatty acid and all micronutrients assessed in this study were significantly lower among participants with the highest adherence to the unhealthful provegetarian FP (G3) compared to participants in G1.

Prevalence of inadequate intake, below the EAR, for each micronutrient and the average number of micronutrients with intakes below the EAR adjusted for sex and age, according to groups of each score is summarised in Table 5. In general, there was a lower prevalence of micronutrient inadequacy (except for vitamin B_12_ and I) in the highest group of the overall provegetarian FP. Among participants with the highest adherence to the healthful provegetarian FP, a higher prevalence of inadequacy was found for vitamins B_1_, B_2_, B_3_, B_12_, Ca, P, Se, I and Zn. Conversely, participants in the highest group of the unhealthful provegetarian FP had the highest prevalence of inadequate dietary intake for every vitamin and mineral. Overall, the lowest prevalence of micronutrient inadequacy was for vitamins B_3_, B_12_ and P, and the highest for folic acid and vitamins D and E.

**Table 5:** Prevalence (%) of failing to meet EAR for each micronutrient and the average number of micronutrients failing to meet EAR according to groups of adherence to the overall, healthful and unhealthful provegetarian FP

	Overall provegetarian FP			Healthful provegetarian FP			Unhealthful provegetarian FP		
	G1	G2	G3	G1	G2	G3	G1	G2	G3
Range	12–35	36–39	40–57	30–52	53–58	59–82	31–55	56–60	61–83
*n*	7032	5452	5341	6295	5670	5860	6745	5310	5770
Number of nutrients below the EAR	3·4	2·8	2·3	2·7	3·0	3·0	1·9	2·8	4·1
Prevalence (%) of failing to meet EAR									
Vitamin A	9·3	5·0	1·8	8·2	5·8	3·1	0·9	3·9	13·2
Vitamin E	97·1	94·7	88·9	94·4	94·7	92·7	90·4	94·9	97·1
Vitamin C	3·3	0·9	0·2	1·9	2·1	1·0	0·2	1·1	3·8
Vitamin B_1_	6·3	2·7	0·9	1·7	4·4	4·8	0·4	2·6	8·2
Vitamin B_2_	2·3	2·1	1·3	0·6	2·2	3·0	0·2	1·1	4·7
Vitamin B_3_	0·2	0·1	0·1	0·03	0·1	0·3	0·0	0·1	0·4
Vitamin B_6_	2·4	0·9	0·3	0·7	1·7	1·6	0·03	0·5	3·6
Vitamin B_12_	0·5	0·9	1·3	0·2	0·6	1·8	0·2	0·6	1·9
Vitamin D	73·6	74·9	73·5	75·3	73·9	72·5	60·6	77·0	86·7
Folic acid	52·2	30·9	13·9	43·1	35·2	23·8	13	33·6	59·7
Fe	2·4	0·6	0·1	0·6	1·5	1·4	0·1	0·7	2·9
Ca	19·9	20·1	18·2	13·5	20·1	25·3	8·7	18·6	32·9
K	19·5	9·1	3·4	11·2	13·3	10·1	2·3	9·1	24·4
Mg	28·9	16·4	7·4	17·4	20·8	17·9	5·2	17·4	35·4
P	0·2	0·2	0·1	0·03	0·2	0·3	0·0	0·04	0·5
Cr	2·7	1·1	0·2	0·9	1·9	1·6	0·1	1·0	3·4
Se	5·4	4·4	3·6	1·9	4·8	7·1	1·1	3·6	9·5
I	6·5	9·4	10·7	5·5	8·5	12·1	4·1	7·7	14·7
Zn	8·2	5·8	3·9	3·4	7·1	8·2	1·5	4·9	12·7

EAR, estimated average requirement; FP, food pattern; G, groups.

**Fig. 1:**
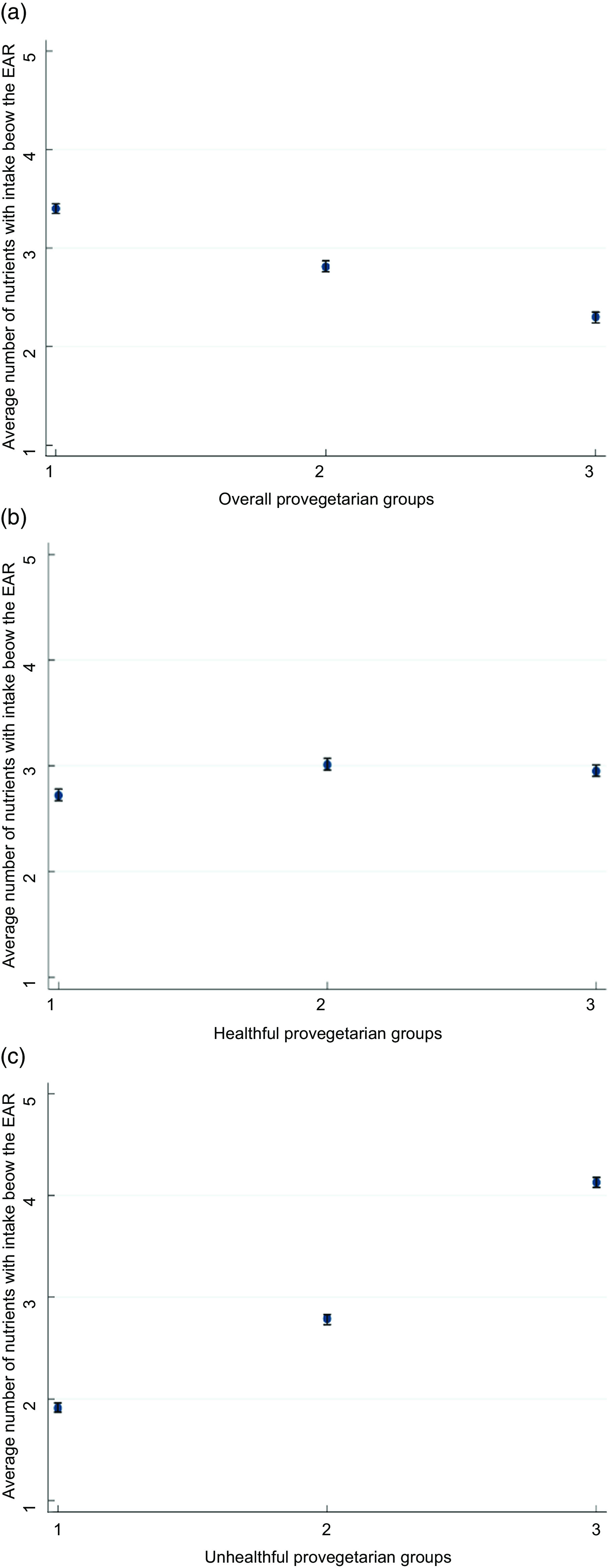
(a) Average number and 95 % CI of micronutrients with intakes below the EAR according to groups of the overall provegetarian FP. Adjusted for sex and age. (b) Average number and 95 % CI of micronutrients with intakes below the EAR according to groups of the healthful provegetarian FP. Adjusted for sex and age. (c) Average number and 95 % CI of micronutrients with intakes below the EAR according to groups of the unhealthful provegetarian FP. Adjusted for sex and age. EAR, estimated average requirement; FP, food pattern

On average, the highest group of the overall provegetarian FP showed the lowest average number (2·3) of micronutrients failing to meet EAR, while the highest group of the unhealthful provegetarian FP exhibited the highest average number (4·1) of micronutrients failing to meet EAR (Figs 1(a) and (c) respectively).

Tables 6 and 7 present the OR of failing to meet EAR for either ≥ 3 or ≥ 6 micronutrients, respectively, according to groups of the different provegetarian FPs. As shown in Table 6, greater adherence to the overall and the healthful provegetarian FP showed an inverse association with the risk of failing to meet ≥ 3 EAR values. The adjusted OR (95 % CI) for failing to meet ≥ 3 EAR (third *v*. first group) was 0·61 (0·54, 0·69), for the overall, 0·27 (0·24, 0·31) for the healthful and 9·04 (7·57, 10·4) for the unhealthful provegetarian FP. Only the healthful and unhealthful provegetarian FPs showed a statistically significant association with the risk of failing to meet ≥ 6 EAR values (Table 7). The adjusted OR (95 % CI) for failing to meet ≥ 6 EAR (third *v*. first group) was 0·31 (0·24, 0·42) for the healthful and 15·00 (8·95, 25·13) for the unhealthful provegetarian FP. Moreover, the analysis of the association between the healthful and unhealthful provegetarian FP and failing to meet EAR values showed a noticeable variation of the estimates after adjusting for age, sex, supplement consumption and total energy intake, which was not observed for the overall provegetarian FP. These results remained substantially unchanged after performing the above-described sensitivity analyses to verify their robustness (data not shown).

**Table 6:** OR (95 % CI) of failing to meet the EAR for ≥ 3 micronutrients according to groups of adherence to the overall, healthful and unhealthful provegetarian FP

	G1	G2		G3		*P*_for trend_
		OR	95 % CI	OR	95 % CI	
**Overall provegetarian FP**						
*n*	7032	5452		5341		
Prevalence of ≥ 3 inadequate micronutrient intake	27·4	18·8		10·5		
Crude	1 (Ref.)	0·61	0·56, 0·67	0·31	0·28, 0·35	< 0·001
Multivariable 1	1 (Ref.)	0·86	0·77, 0·95	0·61	0·54, 0·69	< 0·001
**Healthful provegetarian FP**	G1	G2		G3		
*n*	6295	5670		5860		
Prevalence of ≥ 3 inadequate micronutrient intake	17·8	21·4		20·1		
Crude	1 (Ref.)	1·26	1·15, 1·38	1·17	1·07, 1·28	< 0·001
Multivariable 1	1 (Ref.)	0·54	0·48, 0·60	0·27	0·24, 0·31	< 0·001
**Unhealthful provegetarian FP**	G1	G2		G3		
*n*	6745	5310		5770		
Prevalence of ≥ 3 inadequate micronutrient intake	4·89	17·29		39·2		
Crude	1 (Ref.)	4·06	3·56, 4·63	12·6	11·1, 14·2	< 0·001
Multivariable 1	1 (Ref.)	2·85	2·46, 3·30	9·04	7·57, 10·4	< 0·001

EAR, estimated average requirement; FP, food pattern; G, groups.

Multivariable 1: adjusted for age, sex, supplement consumption (yes/no) and total energy intake (continuous).

**Table 7:** OR (95 % CI) of failing to meet the EAR for ≥ 6 micronutrients according to groups of adherence to the overall, healthful and unhealthful provegetarian FP

	G1	G2		G3		*P*_for trend_
		OR	95 % CI	OR	95 % CI	
**Overall provegetarian FP**						
*n*	7032	5452		5341		
Prevalence of ≥ 6 inadequate micronutrient intake	5·72	3·34		1·54		
Crude	1 (Ref.)	0·57	0·48, 0·68	0·26	0·20, 0·33	< 0·001
Multivariable 1	1 (Ref.)	0·98	0·79, 1·22	0·85	0·64, 1·13	0·305
**Healthful provegetarian FP**	G1	G2		G3		
*n*	6295	5670		5860		
Prevalence of ≥ 6 inadequate micronutrient intake	2·08	4·74		4·54		
Crude	1 (Ref.)	2·34	1·89, 2·89	2·24	1·81, 2·77	< 0·001
Multivariable 1	1 (Ref.)	0·76	0·58, 0·98	0·31	0·24, 0·42	< 0·001
**Unhealthful provegetarian FP**	G1	G2		G3		
*n*	6745	5310		5770		
Prevalence of ≥ 6 inadequate micronutrient intake	0·24	2·00		9·43		
Crude	1 (Ref.)	8·6	5·06, 14·51	43·80	26·6, 72·1	< 0·001
Multivariable 1	1 (Ref.)	3·48	2·00, 6·00	15·0	8·95, 25·13	< 0·001

EAR, estimated average requirement; FP, food pattern; G, groups.

Multivariable 1: adjusted for age, sex, supplement consumption (yes/no) and total energy intake (continuous).

## Discussion

Our findings showed that, as hypothesised, both higher overall and healthful provegetarian FP scores were inversely associated with the risk of micronutrient inadequacy (failing to meet ≥ 3 EAR values). On the contrary, a direct association was found between the unhealthful provegetarian FP and the risk of failing to meet micronutrient requirements. These results could play a useful role in contributing to the development of dietary guidelines based on the importance of nutritional content (i.e. micronutrient adequacy) besides the plant or animal origin of foods.

To the best of our knowledge, this is the first study to evaluate the association between different provegetarian FP with varying degrees of healthiness and the risk of micronutrient inadequacy in an adult population. Other prospective studies have previously examined the relationship between these provegetarian FP indices and several chronic disease^(13,17)^ as well as mortality^(9,31)^.

Diet quality indices, dietary patterns and food-based scores are all valid tools to determine the adequacy of micronutrient intake^(32)^. This focus is relevant due to the increasing prevalence of micronutrient inadequacy across the European general population. A study showed that the prevalence of inadequate intakes was particularly high for vitamins D, C, folic acid, Ca, Se and I in adults^(33)^, which is consistent with our findings. Moreover, the ANIBES study found that there were inadequate intakes for ≥ 3 micronutrients across all age groups in a representative sample of the Spanish population^(34)^. Our results suggest that the adoption of an overall and healthful provegetarian FP could reduce the prevalence of micronutrient inadequacies.

When categorising participants into groups, the highest prevalence of inadequacy was found in the highest group of the unhealthful provegetarian FP compared to lowest group. However, a direct association was found between the adherence to overall provegetarian FP and the risk of micronutrient inadequacy of vitamin B_12_ and I. It has been previously reported that the requirements of these two micronutrients cannot be met in some vegetarian diets^(10,35,36)^. These results are in line with other studies that evaluated the degree of micronutrient adequacy when switching from omnivorous to PBD^(37–42)^. It is also worth noting that in this cohort, participants who had the highest overall provegetarian FP scores were more likely to have prevalent hypertension, CVD, diabetes, dyslipidaemia, cancer and hypercholesterolaemia, which could explain why they followed a special, and generally healthier, diet. Interestingly, the analysis of the association between the healthful and unhealthful provegetarian FP and failing to meet ≥ 6 micronutrients shows a noticeable change of the OR estimate (i.e. attenuated effect) after adjusting for age, sex, supplement consumption and total energy that is not shown for the overall provegetarian FP. These changes could be explained because participants with greater adherence to the healthful and unhealthful provegetarian FP showed lower energy intake and as expected, with higher food consumption, there is a lower risk of failing to meet ≥ 6 EAR values.

Although PBD are widely recognised as a healthy dietary pattern, not every plant food is equally healthy and may exert different health effects due to their nutrient compositions^(43,44)^. These results highlight the importance of taking into account the nutrient density of different kinds of PBD and promoting a healthful provegetarian FP with a high consumption of fruits, vegetables, legumes, nuts and seeds, wholegrain products and olive oil^(13,17,45)^. On the other hand, an unhealthful provegetarian FP includes lower-quality foods such as ultra-processed foods, sweets and desserts, miscellaneous ready-to-eat meals, sugary drinks, fruit juices, refined cereals and red and processed meat^(13)^.

There is currently sufficient evidence that a well-planned healthful provegetarian FP^(10,35,36)^ have many health benefits^(9,13,16,46–48)^, and this could be partly due to its ability to adequately meet the intake of essential nutrients.

We acknowledge that our study has some limitations. First, we used a self-reported FFQ, which can lead to measurement errors and may not be the best method to evaluate the intake of Se, Fe and folic acid^(49)^. However, FFQ is the most practical and feasible tool to evaluate diet in large epidemiological studies^(20,21)^. Second, as in any observational study, some residual confounding might be present. However, we carried out the analyses adjusting for the main known confounders of nutritional adequacy, and we do not consider it as a likely important bias impacting our results. Third, the micronutrient intake may have been underestimated, as we did not calculate the average intake from all food sources. We included the intake from food and dietary supplements, without considering the intake of fortified foods or medication that the participants might be consuming. Fourth, the results based on the EAR cut-point method only estimate the probability of adequacy but does not indicate nutrient deficiencies or whether the diet of this population is actually adequate. Nutritional deficiency should be confirmed by biological markers of nutrient intake. Finally, participants of the SUN cohort cannot be considered representative of the general population, and this could have reduced the variability between subjects in dietary exposures as they belong to a single (high) educational and socio-economic stratum. In this sense, the fact that all participants are university graduates can also be a strength since this allows obtaining a better quality of self-reported information, improving the retention rate and minimising confusion by educational level and, therefore, by socio-economic status^(50)^.

On the other hand, the strengths of the present study are based on the fact that we used data from a well-known Mediterranean cohort with a large sample size and high response rate. Moreover, we adjusted for numerous potential confounders and we used the probabilistic approach and the EAR cut-off approach^(23)^, and in both cases, the results were very similar. Finally, we used a FFQ repeatedly validated in Spain^(20)^.

In conclusion, our findings showed that a greater adherence to an unhealthful provegetarian FP was directly associated with the risk of micronutrient inadequacy. Therefore, PBD do not always lead to a favourable nutritional quality, and it would be advisable that when PBD are followed, they should mostly include nutrient-dense plant foods and minimise ultra-processed and less-healthy plant foods. Our results reinforce the importance of a preference for healthier plant foods in terms of micronutrient intake adequacy in adults.

## Supplementary material

For supplementary material accompanying this paper visit https://doi.org/10.1017/S136898002200204X. click here to view supplementary material
