# Supplementary material for: Healthful and unhealthful provegetarian food patterns and micronutrient intake adequacy in the SUN cohort
Source: Public Health Nutr. 2022 Sep 20;26(3):563–74. doi: 10.1017/S136898002200204X (PMC9989716; doi:10.1017/S136898002200204X)
Supplement: Supplementary file 1 [file S136898002200204Xsup001.pptx]

## Slide 1
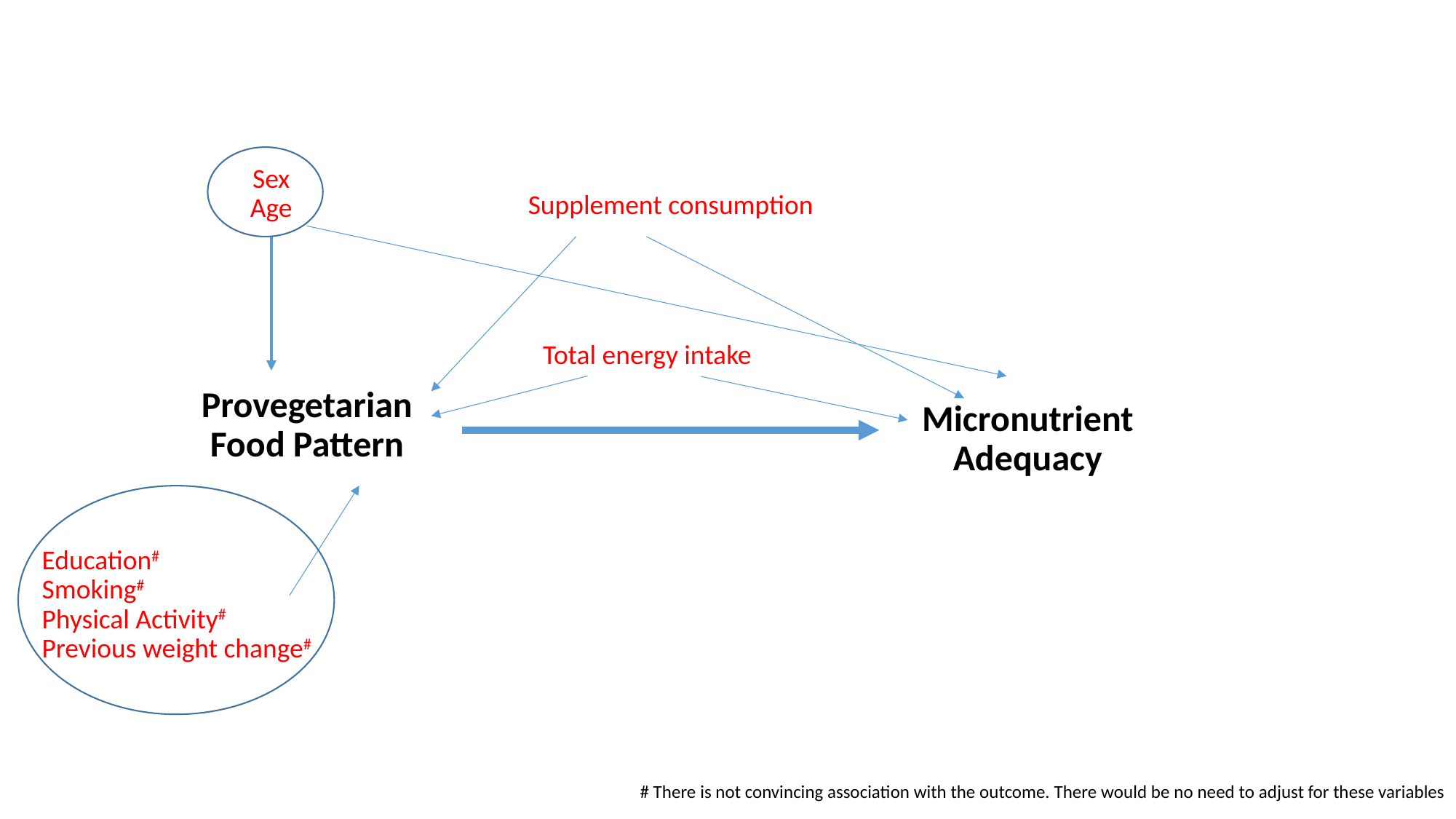

Supplement consumption
Sex
Age
Total energy intake
# Provegetarian Food Pattern
Micronutrient Adequacy
Education#
Smoking#
Physical Activity#
Previous weight change#
# There is not convincing association with the outcome. There would be no need to adjust for these variables
